# A Bedaquiline, Pyrazinamide, Levofloxacin, Linezolid, and Clofazimine Second-line Regimen for Tuberculosis Displays Similar Early Bactericidal Activity as the Standard Rifampin-Based First-line Regimen

**DOI:** 10.1093/infdis/jiad564

**Published:** 2023-12-07

**Authors:** Kayvan Zainabadi, Stalz Charles Vilbrun, Laurent Daniel Mathurin, Kathleen Frances Walsh, Jean William Pape, Daniel W Fitzgerald, Myung Hee Lee

**Affiliations:** Center for Global Health, Weill Cornell Medicine, NewYork, New York; Les Centres GHESKIO, Port-au-Prince, Haiti; Les Centres GHESKIO, Port-au-Prince, Haiti; Center for Global Health, Weill Cornell Medicine, NewYork, New York; Division of General Internal Medicine, Department of Medicine, Weill Cornell Medicine, New York, New York; Center for Global Health, Weill Cornell Medicine, NewYork, New York; Les Centres GHESKIO, Port-au-Prince, Haiti; Center for Global Health, Weill Cornell Medicine, NewYork, New York; Center for Global Health, Weill Cornell Medicine, NewYork, New York

**Keywords:** EBA, MDR, fluoroquinolone, diarylquinoline, treatment shortening

## Abstract

**Background:**

In 2018 the World Health Organization recommended a switch to an all oral bedaquiline-based second-line regimen for treatment of drug-resistant tuberculosis (DR-TB). How these new second-line regimens fare in comparison to first-line regimens for treatment of drug-sensitive tuberculosis (DS-TB) is not well known.

**Methods:**

In this study, we contemporaneously enrolled subjects with DS-TB (n = 31) or DR-TB (n = 23) and assessed their response to therapy with first-line (rifampin, isoniazid, ethambutol, pyrazinamide) or second-line (bedaquiline, pyrazinamide, levofloxacin, linezolid, clofazimine) regimens, respectively.

**Results:**

We found that the early bactericidal activity of first- and second-line regimens was similar during the first 2 weeks of therapy as determined by BACTEC MGIT, colony-forming units, and a liquid limiting dilution assay capable of detecting differentially detectable/culturable *Mycobacterium tuberculosis*. Furthermore, an identical percentage (77.8%) of subjects from the DS-TB and DR-TB cohorts converted to culture negative after 2 months of therapy.

**Conclusions:**

Despite presenting with more advanced disease at time of treatment, subjects with DR-TB receiving an all oral bedaquiline-based second-line treatment regimen displayed a similar microbiological response to therapy as subjects with DS-TB receiving a first-line treatment regimen.

## Teaser

This study reports that the early bactericidal activity of a bedaquiline-based second-line regimen for drug-resistant tuberculosis (TB) is similar to that of the standard rifampin-based first-line regimen for drug-sensitive TB.

In 2021, an estimated 10.6 million individuals were diagnosed with tuberculosis (TB) and 1.6 million died of the disease [1]. Approximately 4% of these infections consisted of drug-resistant TB (DR-TB; defined as rifampin or multidrug resistance), which accounted for a disproportionate 12% of TB-related deaths [1]. DR-TB requires longer treatment regimens with second-line drugs that are more toxic and expensive, which strains public health resources and promotes treatment noncompliance, further driving drug resistance. Troublingly, rates of DR-TB are on the rise globally (up 3.1% from 2020 rates), which poses a threat for TB control and elimination efforts [1].

A major advancement in the treatment of DR-TB came with the approval of bedaquiline, a new diarylquinoline drug that is the current backbone of second-line regimens [2]. Clinical trials have repeatedly demonstrated that addition of bedaquiline to existing second-line regimens improves culture conversion rates and decreases mortality in patients with DR-TB [3–9]. Furthermore, replacement of second-line injectable aminoglycoside drugs with bedaquiline resulted in comparably high treatment success rates but with less toxicity [10–12]. These findings led to the decision in 2018 by the World Health Organization (WHO) to recommend a switch to all oral bedaquiline-based second-line regimens for treatment of DR-TB [13]. How these new second-line regimens fare in comparison to first-line regimens for the treatment of drug-sensitive TB (DS-TB), however, has not been thoroughly studied.

In this study, we contemporaneously enrolled subjects with DS- or DR-TB at the GHESKIO Centers in Port-au-Prince, Haiti, and monitored their response to first- or second-line therapy, respectively. The early bactericidal activity (EBA) of these 2 regimens, along with their corresponding effects on clinical signs and symptoms, during the first 2 months of therapy is presented.

## METHODS

### Study Population and Design

This was a prospective observational study conducted at the Groupe Haïtien d’Étude du Sarcome de Kaposi et des Infectieuses Opportuniste (GHESKIO) Centers in Haiti, and was approved by both the Weill Cornell Medical College and GHESKIO institutional review boards. All participants provided written informed consent.

All aspects about the study design, including exclusion/inclusion criteria and participant characteristics, have been previously reported [14–16]. In short, the following enrollment criteria were used for the DS-TB cohort: ≥18 years of age; diagnosis of active pulmonary TB based on clinical symptoms and signs, chest radiography, and Xpert MTB/RIF (Cepheid, Sunnyvale, California) positivity, without indication of rifampin resistance; no indication of extrapulmonary manifestations of TB; and no history of previous TB treatment. The DR-TB cohort met the same criteria, except participants were not excluded if they had previously been treated for DS-TB; Xpert indicated rifampin resistance; and drug susceptibility testing confirmed resistance to rifampin. Drug susceptibility testing for isoniazid, rifampin, and ethambutol was performed using the BACTEC MGIT SIRE kit and on 7H10 based on standard methods [17], and for pyrazinamide using the BACTEC MGIT Z kit.

Subjects with DS-TB were followed in the GHESKIO outpatient clinic for the duration of their directly observed therapy (DOT). These participants received isoniazid, rifampin, ethambutol, and pyrazinamide for 2 months and then isoniazid and rifampin for 4 months. Subjects with DR-TB were hospitalized in GHESKIO's inpatient multidrug-resistant TB hospital for approximately the first 4 months of therapy with DOT regimens comprised of bedaquiline (400 mg/day for 2 weeks then 200 mg 3 times per week for 22 weeks), levofloxacin (20 mg/kg daily for duration of treatment), linezolid (600 mg/day for 12 months), clofazimine (100 mg/day for duration of treatment), and pyrazinamide (30–40 mg/kg/day for duration of treatment). Of note, bedaquiline was discontinued after 6 months and linezolid after 12 months, with the remaining drugs continued to complete 20 months of therapy. Subjects who demonstrated pyrazinamide resistance continued to receive pyrazinamide for the duration of their therapy. TB signs and symptoms (cough, dyspnea, hemoptysis, fever, and pleuritic chest pain) were graded according to the Division of AIDS grading system [18].

### Sputum Processing and Microbiological Assays

Overnight sputum was collected from both cohorts prior to initiation of therapy, and at 2 weeks and 2 months after initiation of therapy in a cool box with ice packs (4°C). Some sample collections were missed because of the coronavirus disease 2019 (COVID-19) pandemic, some samples resulted in contamination during culture or did not yield enough sputum to perform culture, resulting in a variable number of sputum samples available for analysis at each timepoint.

Experimental work took place in a biosafety level 3 laboratory at GHESKIO with appropriate safety guidelines and personal protective equipment. Decontamination of sputum, preparation of culture filtrate (CF), and colony-forming unit (CFU), limiting dilution (LD), and BACTEC MGIT assays were conducted as previously reported [14, 15, 19, 20]. There are *Mycobacterium tuberculosis* (*Mtb*) populations in human sputum that are not detected by standard culture methods and these *Mtb* are not enumerated by CFU on solid agar [14, 19, 20]. These *Mtb* can be detected using an LD assay (with or without CF) and are called differentially detectable/culturable *Mtb* (DD *Mtb*) [19]. Positivity for DD *Mtb* was defined as previously reported [14, 20], namely when the CFU value was less than the lower bound of the 95% confidence interval (CI) of the most probable number (MPN) estimation from the LD assay with or without CF, which promotes recovery of DD *Mtb* populations from certain sputum samples [14, 15, 20]. LD assays performed with and without CF are referred to as MPN^+CF^ and MPN^−CF^, respectively. To quantify viable *Mtb* from participant sputa, the highest *Mtb* number per milliliter obtained from CFU, MPN^−CF^, or MPN^+CF^ was used (referred to as *Mtb*^Max^). Rate of *Mtb* killing during the first 2 weeks of therapy was only calculated for subjects who had data available at both timepoints. Lack of culture conversion to negative at month 2 was defined as positivity by at least 1 culture method (BACTEC MGIT, CFU, MPN^−CF^, or MPN^+CF^).

### Data Analysis

Data were uploaded to REDCap [21, 22], a secure web-based software platform designed to support data capture for research studies. Data were summarized and analyzed using R software 4.2.2. *Mtb* count obtained by either CFU or MPN-LD assays are presented as log_10_ values per milliliter of sputum. Continuous variables were summarized by median and interquartile range and categorical variables were summarized by count and percentage. Continuous measures were compared using the Mann–Whitney test and categorical measures were compared by the χ^2^ test between groups. Rate of *Mtb* killing during early treatment is defined by the absolute difference in the *Mtb* counts quantified at baseline and after 2 weeks of therapy. For factors associated with continuous measures such as the rate of *Mtb* killing during early treatment, we performed univariate linear regression and reported the regression coefficients and 95% CIs.

## RESULTS

### Study Population

We enrolled 31 subjects with DS-TB and 23 subjects with DR-TB at the GHESKIO Centers in Haiti from 2018 to 2021 (Supplementary Table 1). Subjects with DS-TB received a standard first-line regimen consisting of isoniazid, rifampin, ethambutol, and pyrazinamide for 2 months and then isoniazid and rifampin for 4 months. Subjects with DR-TB received a second-line regimen consisting of bedaquiline, pyrazinamide, levofloxacin, linezolid, and clofazimine for the first 6 months of therapy, with bedaquiline discontinued after 6 months and linezolid after 12 months (for a total of 20 months of therapy).

Subjects with DS-TB were all treatment naive, whereas 82.6% of subjects with DR-TB had previously received treatment for DS-TB (Table 1). All subjects with DR-TB were resistant to rifampin, and 21 of 23 were also resistant to isoniazid (Supplementary Table 1).

**Table 1.** Participant Characteristics for the Drug-Sensitive and Drug-Resistant Tuberculosis Cohorts at Time of Enrollment

Characteristic	DS-TB (n = 31)	DR-TB (n = 23)	*P* Value
Age, y, median (IQR)	31.0 (25.0–39.0)	33.0 (27.5–42.5)	.575
Male sex	19 (61.3)	13 (56.5)	.942
HIV positive	2 (6.5)	4 (17.4)	.408
Xpert positivity			.528
High	13 (41.9)	10 (43.5)	
Medium	12 (38.7)	11 (47.8)	
Low/very low	6 (19.4)	2 (8.7)	
Bilateral disease	9 (30.0)	14 (60.9)	.**049**
Presence of cavities	14 (46.7)	10 (43.5)	1.000
Creatinine, mg/dL, median (IQR)	0.70 (0.60–0.80)	0.60 (0.55–0.70)	.169
Hemoglobin, g/dL, median (IQR)	10.7 (9.57–11.35)	10.6 (9.98–11.45)	.840
Marital status			.585
Married	5 (16.1)	3 (13.0)	
Partnered	7 (22.6)	7 (30.4)	
Single/separated	19 (61.3)	13 (56.5)	
Daily income, USD, median (IQR)	1.18 (0.0–5.36)	1.78 (0.0–6.25)	.887
Highest education			.861
None	4 (12.9)	4 (17.4)	
Primary	8 (25.8)	8 (34.8)	
Secondary	17 (54.8)	9 (39.1)	
University/professional	2 (6.5)	2 (8.7)	
Previously treated for tuberculosis	0 (0.0)	19 (82.6)	**<**.**001**

*P*-values less than 0.05 have been bolded. Data are presented as No. (%) unless otherwise indicated.

Abbreviations: DR-TB, drug-resistant tuberculosis; DS-TB, drug-sensitive tuberculosis; HIV, human immunodeficiency virus; IQR, interquartile range; USD, United States dollars.

### Clinical Response to Therapy

At time of enrollment, subjects with DR-TB had a 2-fold higher rate of bilateral disease (*P* = .049) and a 50% higher rate of dyspnea (*P* = .046) in comparison to subjects with DS-TB (Tables 1 and 2). After 2 months of therapy, the 2 cohorts were comparable in terms of clinical signs and symptoms, except for a modestly higher median pulse oximetry value for subjects with DR-TB (99% for DR-TB vs 98% for DS-TB, *P* = .003) (Table 2, Supplementary Table 2).

**Table 2.** Clinical Characteristics Prior to Therapy and at 2 Months Post–Initiation of Therapy for the Drug-Sensitive and Drug-Resistant Tuberculosis Cohorts

Characteristic	Day 0 (Pretreatment)			Month 2 (Post–Initiation of Therapy)		
	DS-TB (n = 31)	DR-TB (n = 23)	*P* Value	DS-TB (n = 21–24)^a^	DR-TB (n = 19–21)^a^	*P* Value
Dyspnea	18 (58.1)	20 (87.0)	.**046**	12 (50.0)	7 (35.0)	.487
Cough	31 (100.0)	22 (95.7)	.880	19 (79.2)	13 (65.0)	.477
Fever	10 (32.3)	5 (22.7)	.653	0 (0.0)	0 (0.0)	NA
Blood-tinged sputum	0 (0.0)	2 (8.7)	.345	1 (4.2)	1 (5.3)	1.00
Frank hemoptysis	0 (0.0)	0 (0.0)	NA	0 (0.0)	0 (0.0)	NA
Pleuritic chest pain	10 (32.3)	13 (56.5)	.132	10 (41.7)	10 (50.0)	.804
BMI, kg/m^2^, median (IQR)	19.0 (17.3–21.0)	17.9 (17.0–19.6)	.407	19.6 (18.4–21.9)	18.7 (17.4–20.9)	.289
Pulse oximetry, %, median (IQR)	97.00 (95.2–98.0)	97.00 (96.0–98.5)	.386	98.0 (96.0–98.0)	99.0 (97.8–99.0)	.**003**

*P*-values less than 0.05 have been bolded. Data are presented as No. (%) unless otherwise indicated.

Abbreviations: BMI, body mass index; DR-TB, drug-resistant tuberculosis; DS-TB, drug-sensitive tuberculosis; IQR, interquartile range; NA, not applicable; USD, United States dollars.

^a^Not all participant characteristics were recorded for all participants at month 2, resulting in the variable n values.

### Early Bactericidal Activity of First- and Second-line Regimens

**Figure 1.**
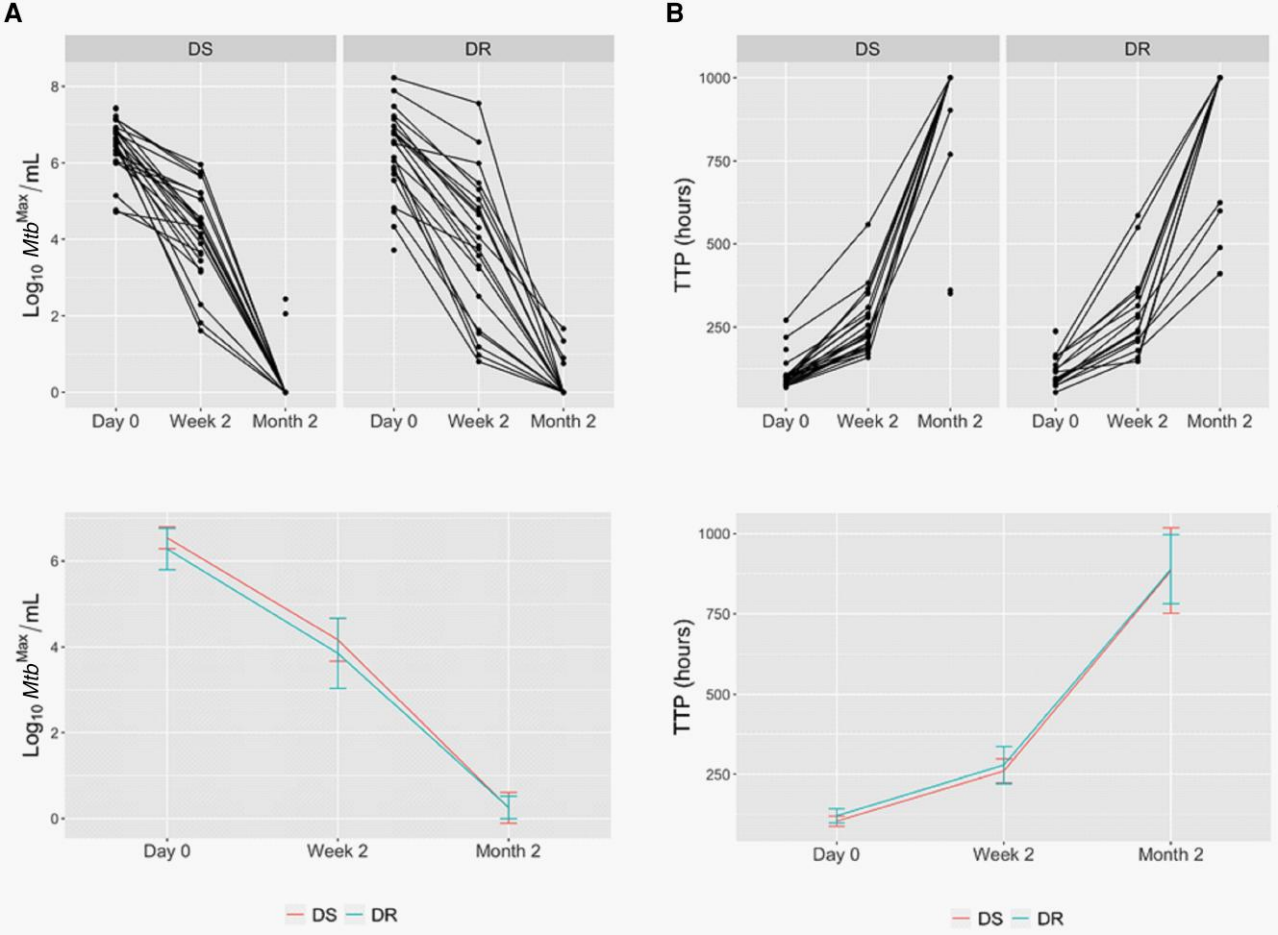
Quantification of viable *Mycobacterium tuberculosis* (*Mtb*) from the sputa of subjects with drug-sensitive (DS) or drug-resistant (DR) tuberculosis during the first 2 months of therapy with first-line or second-line regimens, respectively. *A*, Maximum *Mtb* count (*Mtb*^Max^) obtained by colony-forming units or most probable number from the liquid limiting dilution assay performed with or without culture filtrate. *B*, Time to positivity (TTP) by BACTEC MGIT. Top panels show data per subject and bottom panels show median values per cohort, with error bars representing 95% confidence intervals.

We next assessed the EBA of first and second-line regimens during the first 2 months of therapy. We used 3 assays to quantitate viable *Mtb*: (1) the BACTEC MGIT automated liquid culture system; (2) CFU on solid agar; and (3) liquid LD assay with and without CF that is capable of detecting DD *Mtb*, which are *Mtb* persister populations that fail to grow either on solid medium or in liquid medium unless the suspension has been extensively diluted. Of note, the rate of *Mtb* killing during the first 2 weeks of therapy was similar for first- and second-line regimens as determined by all microbiological assays (Figure 1, Table 3). Furthermore, an identical percentage (77.8%) of subjects from the 2 cohorts converted to culture negative after 2 months of therapy (Supplementary Table 3). Therefore, the 2 regimens exhibit similar bactericidal activity against *Mtb* during the first 2 months of therapy.

**Table 3.** Quantification of Viable *Mycobacterium tuberculosis* (*Mtb*) by Different Microbiological Methods Demonstrates That the Drug-Sensitive and Drug-Resistant Cohorts Display Similar *Mtb* Counts in Their Sputa Prior to Therapy (Day 0) and at 2 Weeks Post–Initiation of Therapy

Method	Day 0 (Pretreatment)			Week 2 (Post–Initiation of Therapy)			Δ (Week 2 vs Day 0)		
	DS-TB (n = 30–31)^a^	DR-TB (n = 22–23)^a^	*P* Value	DS-TB (n = 24–25)^a^	DR-TB (n = 18–22)^a^	*P* Value	DS-TB (n = 24–25)^a^	DR-TB (n = 18–22)^a^	*P* Value
Log_10_ CFU, *Mtb*/mL	6.5 (6.1–6.8)	6.2 (5.2–6.7)	.24	3.6 (3.1–4.4)	3.7 (1.8–4.7)	.73	2.3 (2.0–3.1)	2.4 (2.0–3.2)	.96
Log_10_ MPN^−CF^, *Mtb*/mL	6.6 (6.2–6.9)	6.3 (5.6–6.8)	.21	4.0 (3.2–5.1)	3.7 (1.4–4.7)	.45	2.2 (1.2–3.0)	2.5 (2.1–3.4)	.26
Log_10_ MPN^+CF^, *Mtb*/mL	6.4 (6.1–6.7)	6.5 (5.6–6.9)	.62	4.2 (3.4–5.1)	4.0 (3.2–5.1)	.80	2.0 (1.3–2.8)	2.2 (1.5–3.3)	.41
Log_10_*Mtb*^Max^, *Mtb*/mL^b^	6.7 (6.3–6.9)	6.5 (5.8–6.9)	.33	4.3 (3.6–5.1)	3.9 (2.7–5.0)	.55	2.1 (1.1–2.9)	2.3 (1.8–3.2)	.30
TTP, h	93.5 (79.0–102.5)	105.7 (90.1–152.2)	.13	230.5 (193.0–290.0)	238.0 (210.2–327.5)	.73	148.5 (118.5–178.0)	150.2 (127.6–199.5)	.47

Data are presented as median (interquartile range) unless otherwise indicated.

Abbreviations: CFU, colony-forming units; DR-TB, drug-resistant tuberculosis; DS-TB, drug-sensitive tuberculosis; MPN^–CF^, most probable number from the liquid limiting dilution assay performed without culture filtrate; MPN^+CF^, most probable number from the liquid limiting dilution assay performed with culture filtrate; *Mtb*, *Mycobacterium tuberculosis*; *Mtb*^Max^, maximum *Mycobacterium tuberculosis* count; TTP, time to positivity.

^a^Variable n value at each timepoint is a consequence of contamination events, missed collection timepoints, or insufficient sample quantity to perform microbiological assays.

^b^The maximum *Mtb* count obtained by CFU, MPN^−CF^, or MPN^+CF^.

Multivariate regression analyses did not identify differences in *Mtb* killing during the first 2 weeks of therapy when adjusting for differences in baseline characteristics between the 2 cohorts. However, when limiting analyses only to subjects with bilateral disease, the DR-TB cohort did show improved rates of *Mtb* killing during the first 2 weeks of therapy in comparison to the DS-TB cohort (*P* < .05) (Supplementary Table 4). This difference, however, was not statistically significant when extended to the first 2 months of therapy (Supplementary Table 4).

### Participant Characteristics That Correlate With Culture Conversion at Month 2

**Figure 2.**
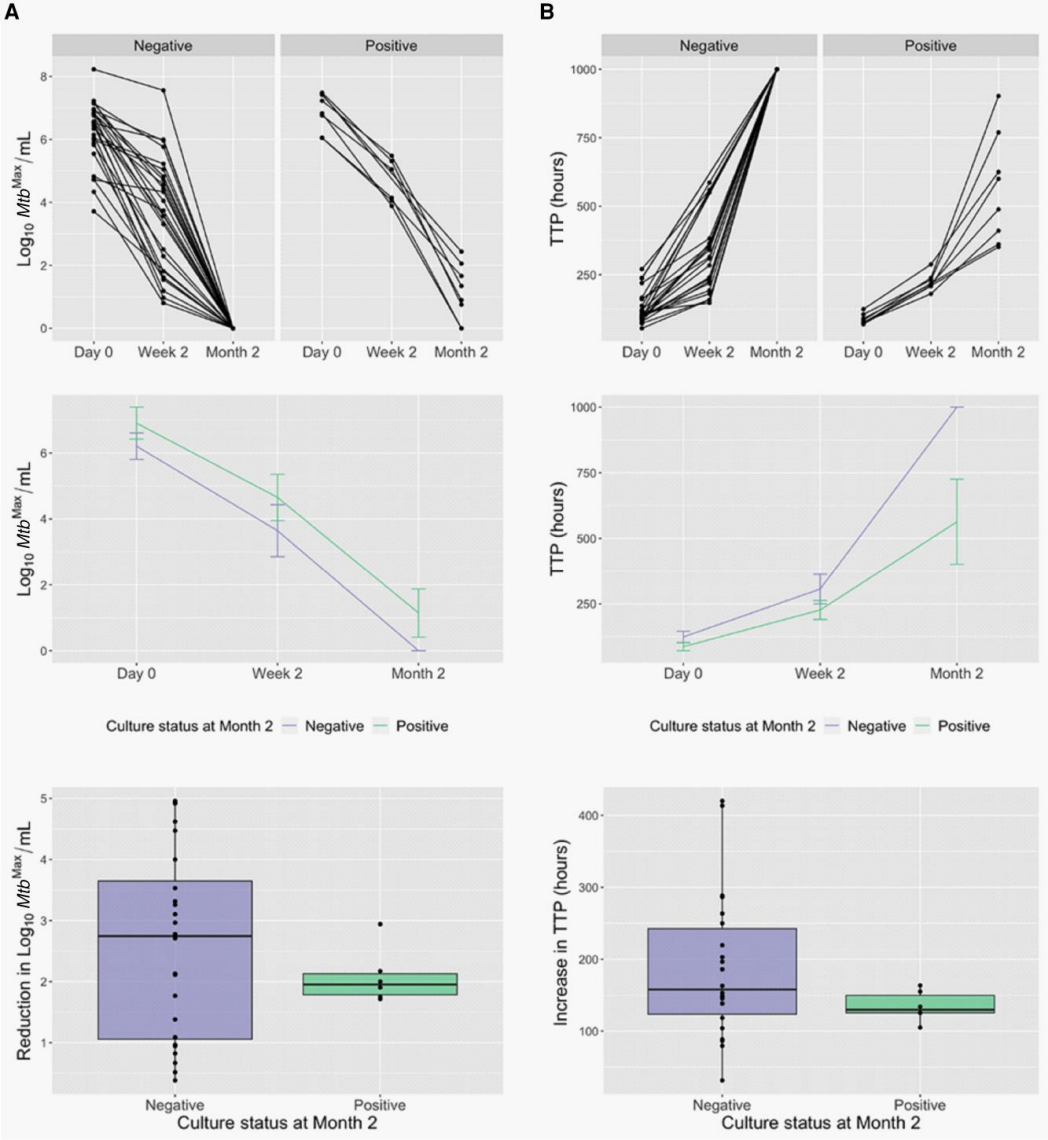
Culture positivity at month 2 is associated with high *Mycobacterium tuberculosis* (*Mtb*) load prior to initiation of therapy as determined by the maximum *Mtb* count (*Mtb*^Max^) obtained by colony-forming units or most probable number from the liquid limiting dilution assay performed with or without culture filtrate (*A*, *P* = .043) or time to positivity (TTP) by BACTEC MGIT (*B*, *P* = .027). However, the rate of *Mtb* killing during the first 2 weeks of therapy is similar for the 2 groups. Top panels show data per subject, middle panels show median values per cohort ± 95% confidence interval, and bottom panels show box plots for the reduction in *Mtb* counts during the first 2 weeks of therapy based on culture status at month 2. Drug-sensitive and drug-resistant cohorts are combined in this analysis.

Next, we examined whether any participant characteristics correlated with lack of culture conversion to negative at month 2. Given the small number of subjects positive by culture at this timepoint, we combined the cohorts for these analyses. Notably, subjects who were culture positive at month 2 all had unresolved dyspnea (*P* < .001) and demonstrated a >20-fold lower gain in weight (*P* = .043) in comparison to subjects who were culture negative (Supplementary Table 5). Consistent with previous reports [23–25], high *Mtb* load (*P* < .05) at time of enrollment significantly correlated with culture positivity at month 2 (Figure 2, Supplementary Table 6), though the rate of *Mtb* killing during the first 2 weeks of therapy did not (Figure 2, Supplementary Table 7). Low BMI (*P* < .05) at time of enrollment and dyspnea (*P* = .001), pleuritic chest pain (*P* = .019), and low BMI (*P* = .013) at 2 months of therapy (Supplementary Tables 8 and 9) also correlated with lack of culture conversion to negative.

### Participant Characteristics That Correlate With Rate of *Mtb* Killing With Therapy

**Figure 3.**
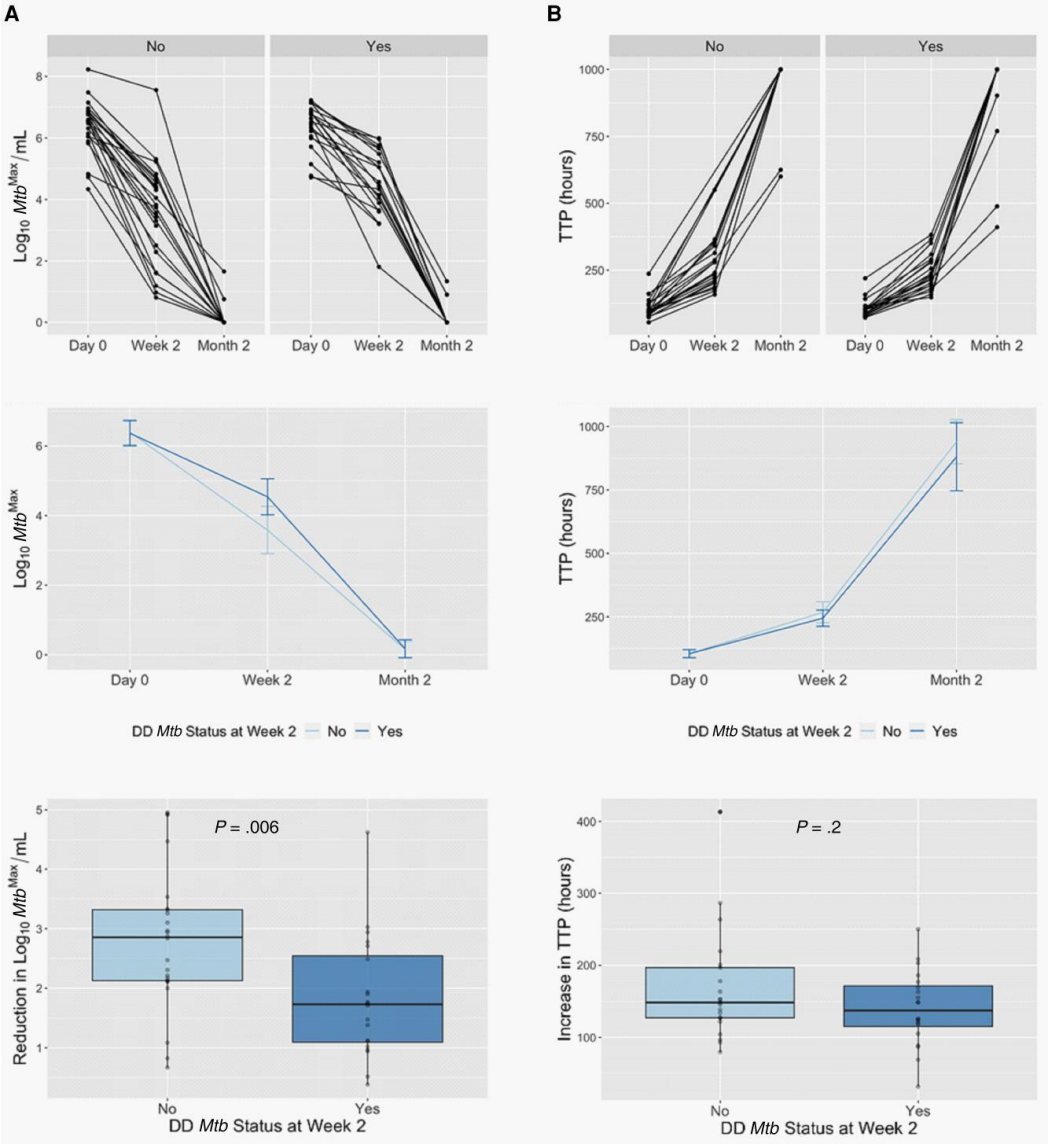
Positivity for differentially detectable/culturable *Mycobacterium tuberculosis* (DD *Mtb*) at week 2 (as determined by the most probable number from the liquid limiting dilution assay performed with culture filtrate [MPN^+CF^] to colony-forming units [CFU] ratio) is associated with a slower rate of *Mtb* killing during the first 2 weeks of therapy as determined by the maximum *Mtb* count (*Mtb*^Max^) obtained by CFU, MPN^−CF^, or MPN^+CF^ (*A*). A similar trend is observed by time to positivity (TTP) by BACTEC MGIT (*B*). Top panels show data per subject, middle panels show median values per cohort ± 95% confidence intervals, and bottom panels show box plots for the reduction in *Mtb* counts during the first 2 weeks of therapy based on DD *Mtb* status.

**Figure 4.**
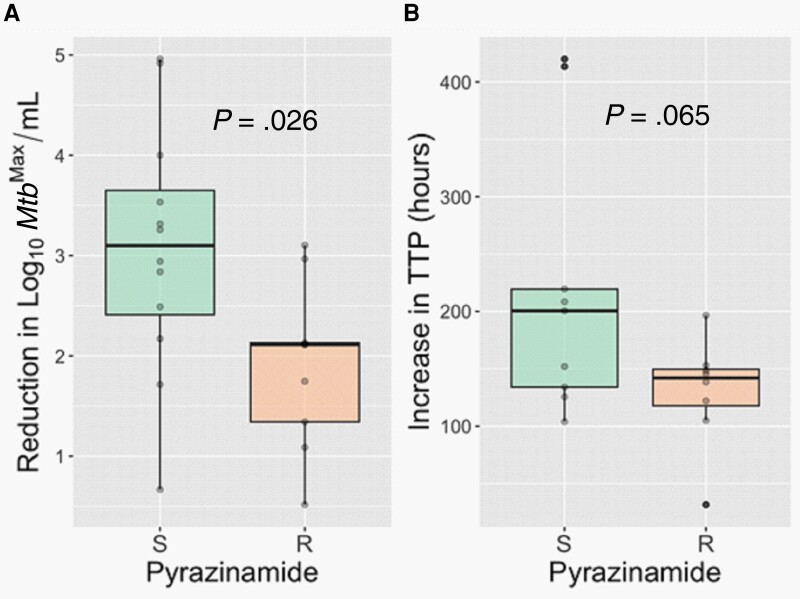
Subjects with drug-resistant tuberculosis who have resistance (R) to pyrazinamide (a drug that is part of their second-line regimen) show a slower rate of *Mycobacterium tuberculosis* (*Mtb*) killing during the first 2 weeks of therapy as determined by the maximum *Mtb* count (*Mtb*^Max^) obtained by colony-forming units or most probable number from the liquid limiting dilution assay performed with or without culture filtrate (*A*) and time to positivity (TTP) by BACTEC MGIT (*B*). Abbreviations: S, sensitive; R, resistant.

Finally, we assessed whether any participant characteristics correlated with the rate of *Mtb* killing during the first 2 weeks of therapy. As before, we combined the cohorts for these analyses. Consistent with a recent report [26], positivity for DD *Mtb* at week 2 correlated with a slower rate of *Mtb* killing during the first 2 weeks of therapy (*P* = .006; Figure 3, Supplementary Table 10). Subjects who were married or living with a partner had higher rates of *Mtb* killing during the first 2 weeks of therapy in comparison to those who were single, divorced, or separated (Supplementary Table 11) (*P* < .05). Finally, in the DR-TB cohort, resistance to pyrazinamide (a drug that is part of their second-line regimen) correlated with a slower rate of *Mtb* killing during the first 2 weeks of therapy (Figure 4;*P* = .026). While the sample size was small, pyrazinamide resistance did not correlate with lack of culture conversion at month 2 (Supplementary Table 12).

## DISCUSSION

The 2018 WHO recommendation of an all oral bedaquiline-based drug regimen for treatment of DR-TB represented a major advancement in TB care [13]. In these new regimens, bedaquiline replaced the injectable aminoglycosides, which decreased toxicity and simplified DR-TB treatment regimens. In the present study, we examined how a bedaquiline-based second-line regimen for DR-TB performed in comparison to the standard rifampin-based first-line regimen for DS-TB.

Our data indicate that the EBA of a second-line regimen consisting of bedaquiline, levofloxacin, linezolid, clofazimine, and pyrazinamide for DR-TB is comparable to a first-line regimen consisting of rifampin, isoniazid, ethambutol, and pyrazinamide for DS-TB (Figure 1). This was evident by similar rates of *Mtb* killing during the first 2 weeks of therapy (Table 3) and identical rates of conversion to culture negative at 2 months of therapy (Supplementary Table 3). These similarities were accompanied by similar signs and symptoms for the 2 cohorts after 2 months of therapy (Table 2, Supplementary Table 2). Therefore, despite the fact that subjects with DR-TB had more advanced disease at time of treatment, and >80% had previously received treatment for DS-TB, they displayed a similar microbiological response to therapy as their DS-TB counterparts.

Our data further indicate that despite its potent sterilizing activity, bedaquiline is likely not solely responsible for these effects. Although receiving a total of 5 drugs, subjects with DR-TB who showed resistance to pyrazinamide had a slower rate of *Mtb* killing during the first 2 weeks of therapy than those who were sensitive (Figure 4). This finding is consistent with studies showing that inclusion of pyrazinamide in other second-line regimens improved culture conversion rates and treatment outcomes only in participants who retained sensitivity to the drug [27–29]. These data provide further support for the utility of drug susceptibility testing for pyrazinamide whenever possible to better tailor DR-TB treatment regimens.

Finally, given the similarities observed for the 2 treatment regimens, it raises the possibility that subjects receiving the second-line regimen examined here may benefit from a shorter course of therapy. A recent trial of a similar regimen used in this study (containing bedaquiline, levofloxacin, linezolid, and pyrazinamide) found a ≥12-month relapse-free cure rate of 75% after 6 months of therapy; similar to what was observed for the older 24-month regimen (70%) that included injectables [30]. This study was limited by small sample size and frequent drug substitutions for linezolid in the intervention arm. Nonetheless, these findings are consistent with bedaquiline's potent EBA—comparable to that of isoniazid and rifampin [31]—and recent studies showing that the combination of bedaquiline with pyrazinamide and a fluoroquinolone acts as one of the most powerful therapeutic backbones for TB [29, 32, 33]. These findings, however, need to be tempered with rising rates of pyrazinamide resistance (41% found in this study), which was associated with decreased EBA despite the fact that 4 other drugs were coadministered (Figure 4). Recent trials indicate that bedaquiline-based regimens may also hold promise for shortening treatment regimens for DS-TB [29, 34]. While this study was not powered to assess treatment outcomes, future studies examining whether pyrazinamide resistance, or shorter-course therapy with the second-line regimen examined here, are associated with differential clinical outcomes for DR-TB is warranted.

## Supplementary Data

Supplementary materials are available at *The Journal of Infectious Diseases* online (http://jid.oxfordjournals.org/). Supplementary materials consist of data provided by the author that are published to benefit the reader. The posted materials are not copyedited. The contents of all supplementary data are the sole responsibility of the authors. Questions or messages regarding errors should be addressed to the author.

## Supplementary Material

jiad564_Supplementary_Data

## Notes

***Acknowledgments.*** The authors are grateful to the subjects who volunteered for this study, as well as the clinical, research, and administrative staff of GHESKIO who made this study possible. We thank the Foundation Mérieux for helping to build and maintain the biosafety level 3 facility at GHESKIO.

***Author contributions.*** Conceptualization, funding acquisition, and methodology: K. Z., K. F. W., and D. W. F. Supervision and project administration: S. C. V., L. D. M., K. F. W., and J. W. P. Investigation: K. Z. Formal analysis: K. Z. and M. H. L. Writing of original draft, review, and editing: All authors.

***Financial support*.** This work was supported by the National Institutes of Health (grant numbers U19AI111143 and K24AI098627 to D. F. W.). K. Z. was supported by a Vanderbilt-Emory-Cornell-Duke Global Health Fellowship, funded by the Fogarty International Center of the National Institutes of Health (grant number D43 TW009337). Funding to pay the Open Access publication charges for this article was provided by Weill Cornell Medical College.
