# Supplementary material for: A Bedaquiline, Pyrazinamide, Levofloxacin, Linezolid, and Clofazimine Second-line Regimen for Tuberculosis Displays Similar Early Bactericidal Activity as the Standard Rifampin-Based First-line Regimen
Source: J Infect Dis. 2023 Dec 7;230(2):e447–56. doi: 10.1093/infdis/jiad564 (PMC11326837; doi:10.1093/infdis/jiad564)
Supplement: jiad564_Supplementary_Data [file jiad564_supplementary_data.zip › Supp Tables 2-12 (CLEAN) 11-20-2023.docx]

**Table S1. Excel file with all raw data pertaining to participant characteristics and viable Mtb counts by microbiological assays for the DS and DR cohorts.**

**Table S2. Subjects with DS or DR TB show similar changes in clinical signs and symptoms during the first two months of therapy with first-line or second-line regimens, respectively.**

|  | **DS (21-27)^1^** | **DR (n=19-22)^1^** | **p** |
| --- | --- | --- | --- |
| Dyspnea, n (%): |  |  | 0.239 |
| Not Present | 4 (16.7) | 2 (10.5) |  |
| Improved Dyspnea | 8 (33.3) | 11 (57.9) |  |
| Newly Developed Dyspnea | 3 (12.5) | 0 ( 0.0) |  |
| Persistent Dyspnea | 9 (37.5) | 6 (31.6) |  |
| Persistent Cough, n (%) | 19 (79.2) | 12 (63.2) | 0.412 |
| Improved Fever (%) | 6 (22.2) | 5 (22.7) | 1.000 |
| Pleuritic chest pain, n (%): |  |  | **0.058** |
| Not Present | 11 (45.8) | 5 (25.0) |  |
| Improved | 3 (12.5) | 5 (25.0) |  |
| Newly Developed | 8 (33.3) | 3 (15.0) |  |
| Persistent | 2 ( 8.3) | 7 (35.0) |  |
| ∆Weight, kg (median [IQR]) | 2.00 [0.50, 4.00] | 1.60 [0.00, 3.14] | 0.531 |
| ∆Pulse Oximetry, % (median [IQR]) | 1.00 [0.00, 2.00] | 1.00 [0.00, 1.25] | 0.947 |

**^1^** Not all data available for all subjects which results in variable n-value

**Table S3. Culture positivity at two months of therapy (as determined by positivity by either CFU, MPN^-CF^, MPN^+CF^, or BACTEC MGIT) is identical between the two cohorts.**

|  | **DS (n=18)** | **DR (n=18)** | **p** |
| --- | --- | --- | --- |
| Culture positive at 2 months, n (%) | 4 (22.2) | 4 (22.2) | 1.00 |

**Table S4. When limited only to subjects with bilateral disease, the rate of Mtb killing during the first two weeks of therapy was greater for subjects with DR TB receiving the second line regimen in comparison to those with DS TB receiving the first line regimen.** This association did not hold when expanding to the first two months of therapy.

|  |  | | **Positive for bilateral disease** | |  |
| --- | --- | --- | --- | --- | --- |
|  |  | | **DS** | **DR** | **p** |
|  | | n | 9 | 14 |  |
| **Day 0 – Week 2** | | Reduction in log_10_Mtb^Max^/mL (median [IQR]) | 1.25 [1.09, 2.24] | 2.66 [2.11, 3.22] | **0.017** |
|  |  | Increase in TTP, hours (median [IQR]) | 94.75 [88.00, 129.75] | 156.00 [131.88, 202.50] | **0.010** |
| **Day 0 – Month 2** | | Reduction in log_10_Mtb^Max^/mL (median [IQR]) | 5.99 [5.69, 6.57] | 5.89 [5.84, 6.55] | 0.697 |
|  |  | Increase in TTP, hours (median [IQR]) | 896.50 [589.25, 912.25] | 851.75 [507.38, 882.62] | 0.612 |

**Table S5. Changes in clinical characteristics during the first two months of therapy and their association with culture positivity at Month 2 of therapy.** DS and DR cohorts are combined in this analysis.

| **Culture status at Mo 2:** | **Negative (n=28)** | **Positive (n=8)^1^** | **p** |
| --- | --- | --- | --- |
| Persistent Dyspnea, n (%) | 4 (16.0) | 8 (100.0) | **<0.001** |
| Persistent Cough, n (%) | 16 (64.0) | 8 (100.0) | 0.125 |
| Pleuritic chest pain, n (%): |  |  | **0.051** |
| Not Present | 12 (48.0) | 1 (12.5) |  |
| Improved chest pain | 5 (20.0) | 0 (0.0) |  |
| Newly Developed chest pain | 4 (16.0) | 3 (37.5) |  |
| Persistent chest pain | 4 (16.0) | 4 (50.0) |  |
| ΔWeight, kg (median [IQR]) | 2.0 [0.70, 4.3] | 0.10 [-0.9, 2.2] | **0.043** |
| ΔPulse Oximetry, % (median [IQR]) | 0.50 [0.00, 1.75] | 1.00 [0.75, 1.25] | 0.357 |

^1^ Consisting of four participants from each cohort.

**Table S6. High Mtb load prior to initiation of therapy (Day 0) positively correlates with culture positivity at two months of therapy as determined by the maximum Mtb count (Mtb^Max^) obtained by CFU, MPN^-CF^ or MPN^+CF^ and time to positivity (TTP) by BACTEC MGIT .** DS and DR cohorts are combined in this analysis.

| **Culture status at Mo 2:** | **Negative (n=28)** | **Positive (n=8)^1^** | **p** |
| --- | --- | --- | --- |
| Log_10_CFU at Day 0, Mtb/mL (median [IQR]) | 6.0 [5.4, 6.6] | 6.8 [6.3, 7.2] | 0.091 |
| Log_10_MPN^-CF^ at Day 0, Mtb/mL (median [IQR]) | 6.3 [5.8, 6.8] | 7.0 [6.4, 7.4] | 0.054 |
| Log_10_MPN^+CF^ at Day 0, Mtb/mL (median [IQR]) | 6.4 [5.8, 6.7] | 6.8 [6.3, 7.1] | 0.167 |
| Log_10_Mtb^Max^ at Day 0, Mtb/mL (median [IQR]) | 6.5 [5.8, 6.8] | 7.0 [6.6, 7.4] | **0.043** |
| TTP at Day 0, hours (median [IQR]) | 100.7 [90.2, 142.4] | 79.0 [74.6, 93.7] | **0.027** |

^1^ Consisting of four participants from each cohort

**Table S7. The rate of killing of Mtb during the first two weeks of therapy does not significantly correlate with culture conversion to negative at month two of therapy.** DS and DR cohorts are combined in this analysis.

| **Culture status at Mo 2:** | **Negative (n=28)** | **Positive (n=8)^1^** | **p** |
| --- | --- | --- | --- |
| ΔLog_10_CFU, Mtb/mL (median [IQR]) | -2.92 [1.64, 3.58] | -2.19 [2.10, 2.40] | 0.568 |
| ΔLog_10_MPN^-CF^, Mtb/mL (median [IQR]) | -2.85 [1.16, 4.00] | -2.12 [1.92, 2.29] | 0.517 |
| ΔLog_10_MPN^+CF^, Mtb/mL (median [IQR]) | -2.38 [1.30, 3.55] | -1.77 [1.57, 1.88] | 0.614 |
| ΔLog_10_Mtb^Max^, Mtb/mL (median [IQR]) | -2.74 [1.06, 3.65] | -1.95 [1.79, 2.13] | 0.468 |
| ΔTTP, hours (median [IQR]) | 158.0 [123.5, 242.4] | 129.75 [125.5, 149.7] | 0.263 |

^1^ Consisting of four participants from each cohort

**Table S8. Clinical characteristics and their association with culture positivity at Month 2 of therapy.** DS and DR cohorts are combined in this analysis.

|  | **Day 0** | | | | **Month 2** | | | |
| --- | --- | --- | --- | --- | --- | --- | --- | --- |
| **Culture status at Mo 2:** | **Negative (n=28)** | **Positive (n=8)^1^** | **p** | | **Negative (n=25)** | | **Positive (n=8)^1^** | **p** |
| Dyspnea, n (%) | 19 (67.9) | 8 (100.0) | 0.165 | | 6 (24.0) | | 8 (100.0) | **0.001** |
| Cough, n (%) | 27 (96.4) | 8 (100.0) | 1.00 | | 16 (64.0) | | 8 (100.0) | 0.125 |
| Fever, n (%) | 6 (21.4) | 2 (28.6) | 1.00 | | 0 (0) | | 0 (0) | NA |
| Blood-tinged sputum, n (%) | 1 (3.6) | 1 (12.5) | 0.923 | | 2 (8.0) | | 0 (0.0) | 1.00 |
| Frank hemoptysis, n (%) | 0 (0.0) | 0 (0.0) | NA | | 0 (0.0) | | 0 (0.0) | NA |
| Pleuritic chest pain, n (%) | 10 (35.7) | 4 (50.0) | 0.749 | | 8 (32.0) | | 7 (87.5) | **0.019** |
| BMI (median [IQR]) | 19.0 [17.8, 20.5] | 16.8 [15.3, 17.9] | | **0.018** | 19.6 [18.9, 22.5] | 17.2 [16.0, 17.9] | | **0.012** |
| Pulse Oximetry, % (median [IQR]) | 98.0 [96.5, 98.0] | 95.0 [95.0, 97.2] | | 0.109 | 98.0 [98.0, 99.0] | 97.0 [96.0, 98.2] | | **0.082** |

^1^ Consisting of four participants from each cohort; Additional baseline characteristics can be found in Table S5

**Table S9.** **Baseline participant characteristics and their association with culture positivity at two months of therapy.** DS and DR cohorts are combined in this analysis.

| **Culture status at Mo 2:** | **Negative (n=28)** | **Positive (n=8)^1^** | **p** |
| --- | --- | --- | --- |
| Male, n (%) | 19 (67.9) | 5 (62.5) | 1.000 |
| Age in yrs, median [IQR] | 30.50 [25.0, 37.0] | 33.00 [31.7, 36.2] | 0.277 |
| HIV^+^, n (%) | 3 (10.7) | 1 (12.5) | 1.000 |
| Bilateral disease, n (%) | 9 (33.3) | 5 (62.5) | 0.285 |
| Cavities, n (%) | 11 (40.7) | 5 (62.5) | 0.496 |
| Creatinine Result, mg/dl (median [IQR] | 0.70 [0.60, 0.80] | 0.65 [0.58, 0.72] | 0.656 |
| Hemoglobin, g/dl (median [IQR]) | 10.80 [10.25, 11.72] | 9.12 [8.74, 11.0] | 0.065 |
| Married/Partner, n (%) | 11 (39.3) | 4 (50.0) | 0.892 |
| Daily income, USD (median [IQR]) | 0.98 [0.0, 7.14] | 2.39 [0.66, 4.02] | 0.532 |
| Education, secondary or more, n (%) | 18 (64.3) | 4 (50.0) | 0.749 |
| GeneXpert High, n (%) | 8 (28.6) | 4 (50.0) | 0.479 |
| Treated for tuberculosis before, n (%) | 12 (42.9) | 4 (50.0) | 1.000 |

^1^ Consisting of four participants from each cohort

**Table S10. Positivity for DD Mtb at Week 2 as determined by the presence of culture filtrate (CF) in the limiting dilution assay (MPN^+CF^)** **is associated with a slower rate of Mtb killing during the first two weeks of therapy as assessed by the change in the maximum Mtb count (Mtb^Max^) obtained by CFU, MPN^-CF^ or MPN^+CF^ at Day 0 vs Week 2.** Of note, CF has previously been shown to help recovery of DD Mtb populations in post-initiation of therapy samples [14, 20].

|  |  | **Decrease in Mtb^Max^/mL^1^** | | | | **Increase in TTP (hours)** | | | |
| --- | --- | --- | --- | --- | --- | --- | --- | --- | --- |
| **Timepoint DD Mtb assessed:** | **DD Mtb positivity as determined by:** | **n^2^** | **β ^3^** | **95% CI** | **p** | **n^2^** | **β^3^** | **95% CI** | **p** |
| **Day 0 (pre-therapy)** | **MPN^-CF^**/**CFU** | 44 | 0.34 | -0.64, 1.3 | 0.5 | 41 | 27 | -36, 89 | 0.4 |
|  | **MPN^+CF^**/**CFU** | 44 | -0.12 | -1.2, 0.99 | 0.8 | 41 | -20 | -97, 57 | 0.6 |
| **Week 2 (post-initiation of therapy)** | **MPN^-CF^**/**CFU** | 44 | -0.53 | -1.3, 0.22 | 0.2 | 41 | 9.1 | -34, 52 | 0.7 |
|  | **MPN^+CF^**/**CFU** | 44 | -0.99 | -1.7, -0.30 | **0.006** | 41 | -29 | -70, 13 | 0.2 |

^1^ As determined by the maximum Mtb/mL count obtained by CFU, MPN^-CF^, or MPN^+CF^; ^2^ Cohorts combined for this analysis; ^3^ Regression coefficient; D0, day 0; W2, week 2; CF, culture filtrate

**Table S11. Participant characteristics and their association with Mtb killing during the first two weeks of therapy as assessed by the maximum Mtb count (Mtb^Max^) obtained by CFU, MPN^-CF^ and MPN^+CF^ assays or by time to positivity (TTP) by BACTEC MGIT.**

|  | **Decrease in Mtb^Max^/mL^1^** | | | | **Increase in TTP (hours)** | | | |  |
| --- | --- | --- | --- | --- | --- | --- | --- | --- | --- |
| **Characteristic** | **n** | **β ^2^** | **95% CI** | **p** | **n** | **β^2^** | **95% CI** | **p** |  |
| Gender, male | 46 | 0.09 | -0.66, 0.85 | 0.8 | 43 | 5.6 | -45, 57 | 0.8 |  |
| Age | 46 | 0.01 | -0.03, 0.04 | 0.7 | 43 | -0.91 | -3.3, 1.4 | 0.4 |  |
| HIV+ | 46 | 0.49 | -0.58, 1.6 | 0.4 | 43 | 44 | -31, 120 | 0.2 |  |
| Bilateral disease, yes | 45 | 0.06 | -0.69, 0.81 | 0.9 | 42 | -20 | -71, 30 | 0.4 |  |
| Cavities present, yes | 45 | -0.06 | -0.81, 0.69 | 0.9 | 42 | -0.02 | -51, 51 | >0.9 |  |
| Creatinine Result | 46 | 1.1 | -1.0, 3.1 | 0.3 | 43 | -28 | -173, 117 | 0.7 |  |
| Hemoglobin Level | 46 | 0.17 | -0.08, 0.41 | 0.2 | 43 | 3.0 | -15, 21 | 0.7 |  |
| Past TB treatment, yes | 46 | 0.19 | -0.57, 0.94 | 0.6 | 43 | 5.2 | -47, 58 | 0.8 |  |
| Married/Partner | 46 | 0.76 | 0.05, 1.5 | **0.036** | 43 | 57 | 9.6, 104 | **0.019** |  |
| Daily income | 46 | 0.00 | 0.00, 0.00 | 0.8 | 43 | 0.00 | -0.02, 0.02 | 0.8 |  |
| Education, Secondary or more | 46 | -0.14 | -0.88, 0.60 | 0.7 | 43 | 18 | -32, 67 | 0.5 |  |
| Dyspnea, yes | 46 | -0.24 | -1.0, 0.55 | 0.5 | 43 | -31 | -83, 21 | 0.2 |  |
| Cough, yes | 46 | -0.96 | -3.4, 1.5 | 0.4 | 43 | -57 | -220, 106 | 0.5 |  |
| Fever, yes | 45 | -0.45 | -1.3, 0.36 | 0.3 | 42 | -0.36 | -56, 56 | >0.9 |  |
| Pleuritic chest pain, yes | 46 | 0.50 | -0.22, 1.2 | 0.2 | 43 | 4.1 | -46, 54 | 0.9 |  |
| BMI | 44 | 0.04 | -0.10, 0.18 | 0.6 | 41 | 1.0 | -8.4, 10 | 0.8 |  |
| Pulse Oximetry | 45 | 0.04 | -0.15, 0.23 | 0.7 | 42 | 3.0 | -9.9, 16 | 0.6 |  |
| GeneXpert High | 46 | -0.40 | -1.1, 0.35 | 0.3 | 43 | -22 | -73, 28 | 0.4 |  |

^1^ As determined by the maximum Mtb/mL count obtained by CFU, MPN^-CF^, or MPN^+CF^. ^2^ Regression coefficient

**Table S12. Although the sample size was small, pyrazinamide resistance (R) was not associated with lack of culture conversion at month two for the DR cohort receiving a bedaquiline based second line regimen that included pyrazinamide.**

|  | **Pyrazinamide status** | |
| --- | --- | --- |
|  | **R (N=9)** | **S (N=13)** |
| **Culture Month 2:** |  |  |
| Negative | 6 (66.7%) | 8 (61.5%) |
| Positive | 1 (11.1%) | 2 (15.4%) |
| Missing | 2 (22.2%) | 3 (23.1%) |
